# Salmonella Urinary Tract Infection Heralding Thoracic Mycotic Aneurysm: Case Report as Medical Apology

**DOI:** 10.5811/cpcem.2016.11.31538

**Published:** 2017-01-24

**Authors:** Jennifer L. White, Gabriel R. Golfus, Annie T. Sadosty

**Affiliations:** * Mayo Clinic, Department of Emergency Medicine, Rochester, Minnesota; † Mayo Clinic, Pharmacy Services, Rochester, Minnesota

## Abstract

We report a case as a patient apology as a means of teaching other physicians about a unique presentation of a rare disease. *Salmonella* species are unusually isolated organisms in urine. In the case described, appreciation for the rarity of *Salmonella* species in the urine facilitated recognition of a serious disseminated *Salmonella* infection. Physicians should consider disseminated *Salmonella* infection, as was found in a patient with an aortic mycotic an eurysm, after isolation of *Salmonella* in urine despite an initially benign clinical presentation.

## CASE REPORT

A 71-year-old man presented to an outside emergency department (ED) with chief concerns of abdominal pain and anorexia that had been present for four weeks. Findings of an extensive work-up were subsequently unrevealing, including normal results of colonoscopy, esophagogastroduodenoscopy, colon biopsy, mesenteric Doppler studies, and computed tomography (CT) of the abdomen and pelvis. Video capsule endoscopy demonstrated a questionable adynamic ileus. Treatment with dicyclomine hydrochloride, polyethylene glycol 3350, tramadol, and simethicone had failed to relieve symptoms. The patient’s family decided to sign him out of the outside hospital from the inpatient service and drive him directly to our ED for evaluation in the middle of the night.

On initial presentation to our ED, the patient complained of left-sided abdominal pain described as aching and one episode of hematuria. Past medical history included osteoarthritis, hypertension, chronic obstructive pulmonary disease, coronary artery disease, nonischemic cardiomyopathy, chronic atrial fibrillation, embolic stroke, and mediastinal thymoma with sternotomy and subsequent pleural effusion and empyema. Initial findings were as follows: temperature 36.6°C oral, blood pressure 142/95 mm Hg, pulse 106 beats per minute, oxygen saturation 98% while breathing room air, and respiratory rate of 16 breaths per minute. Physical examination findings and results of laboratory analysis were reassuring except for an international normalized ratio of 5.5 and evidence of hemoconcentration. The patient was given intravenous (IV) fluids and 1,000 mg of acetaminophen and was then dismissed. Results of urine culture were positive for growth of more than 100,000 colony-forming units of *Salmonella* species 72 hours later.

Per protocol, the positive urine culture results were flagged and presented to the physician working clinically. On the basis of the patient’s prior presentation and the rarity of *Salmonella* in the urine, the patient was asked to return immediately for reevaluation. On return to the ED, the patient’s wife expressed sincere confusion and disappointment as she tried to explain how her husband, a hardworking man, was told to “walk it off” and that his irritable bowel syndrome was “just something he had to learn to live with.” The patient appeared pale and acutely ill with closed eyes and persistent moaning. Findings at presentation were temperature 37.2°C oral, blood pressure 102/55 mm Hg, pulse 93 beats per minute, oxygen saturation 95% while breathing room air, and respiratory rate of 22 breaths per minute. Pain was rated as 10 on a scale of 0 to 10. Positive physical examination findings included flat neck veins, dry mucous membranes, healed scars on the chest, and left lower quadrant abdominal pain without tenderness or peritoneal signs, inguinal tenderness, or adenopathy. Sepsis work-up and blood cultures were ordered, as was CT because of concern for possible disseminated infection. The patient was given 1 gram of ceftriaxone, IV fluids, and hydromorphone.

**Image 1:**
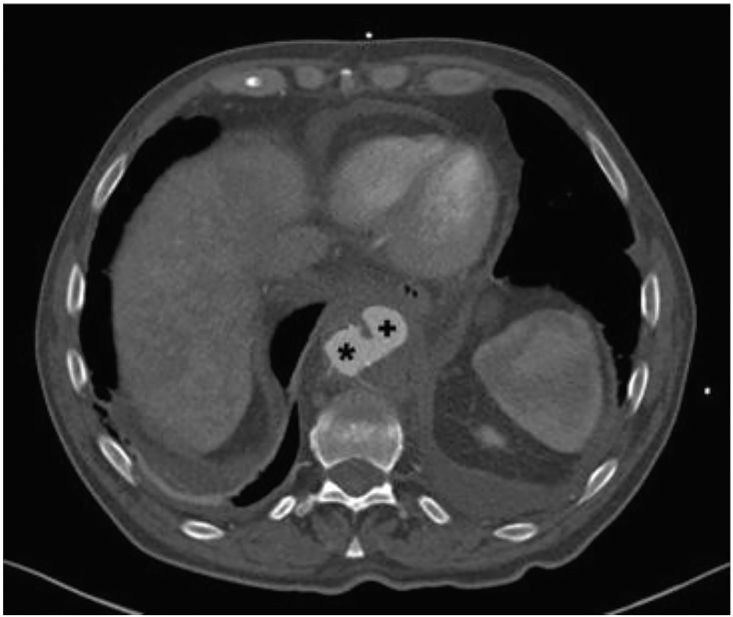
Computed tomogram of pseudoaneurysm (+) in descending thoracic aorta (*).

**Image 2:**
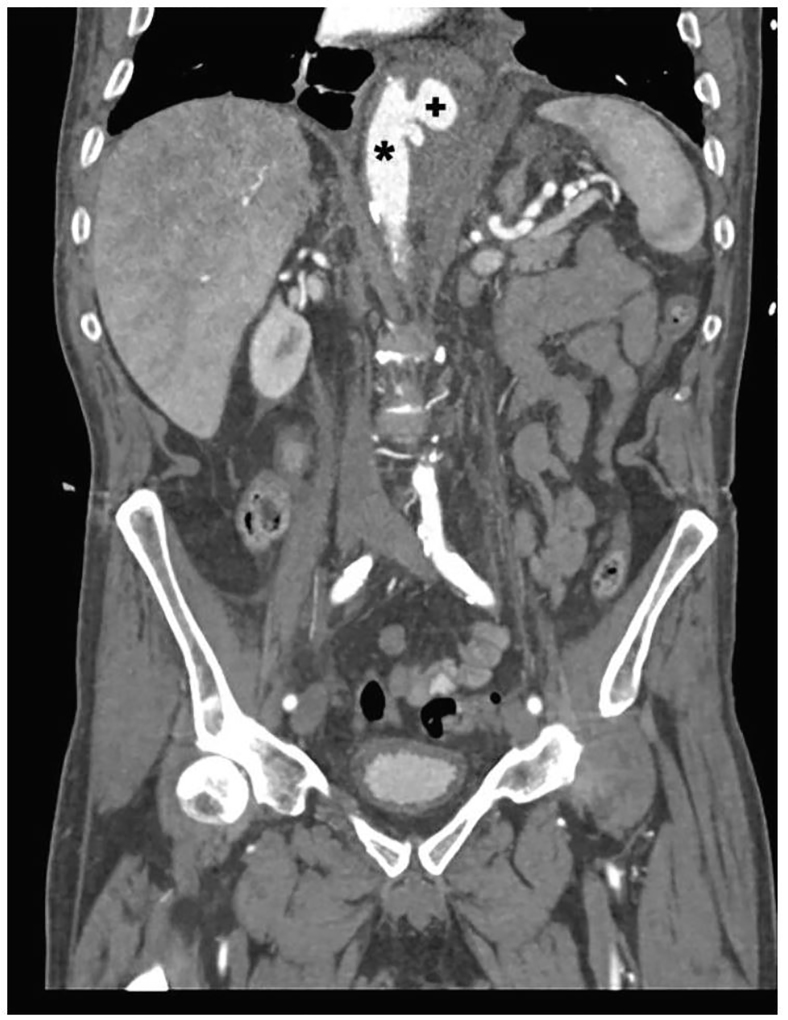
Coronal computed tomogram of pseudoaneurysm (+) in descending thoracic aorta (*).

CT showed a contained pseudoaneurysm in the descending thoracic aorta (4.7×4.0×5.7 cm) about 2 cm in diameter from the native aortic wall to the outer edge of the outpouching; it had a bilobed appearance and a total craniocaudal measurement of 2.6 cm for both outpouchings (Images 1 and 2). The inferior-most origin of the pseudoaneurysm was noted about 3 cm above the origin of the celiac artery. Surrounding hematoma was seen on the descending thoracic aorta with inflammation and “old” hematoma settling down into the diaphragm.

The patient was admitted to a vascular surgery service, and IV ceftriaxone therapy was continued before surgical resection and replacement of a segment of the descending thoracic aorta with a cryopreserved aortic interposition graft. After a two-week hospital stay, the patient was dismissed receiving outpatient IV antibiotic infusion therapy.

## DISCUSSION

*Salmonella* is an unusual organism in the urine; in fact, it accounts for less than 0.07% of all isolates.^1^ *Salmonella* organisms isolated from the urine can result from fecal contamination. A previous case series of urinary tract infections with *Salmonella* isolation suggested that urinary tract infections caused by *Salmonella* isolation in the urine did not substantially differ from typical urinary tract infections except that they could be more severe in patients who had received renal transplants.^2^ However, additional case reports have primarily been related to the association between *Salmonella* bacteriuria and urinary tract anatomical malformations or have suggested the presence of other serious invasive illness.^3^,^4^

In the case described, the patient had salmonellosis with bacteremic seeding of the aorta (aortitis) and persistent bacteremia. The anorexia, left lower quadrant pain, and generalized lethargy that ensued over weeks resulted from persistent bacteremia and also explained his urinary tract infection. Mycotic aneurysm is a misnomer because it is often the result of bacteria. *Salmonella* infection is a common cause of a mycotic aneurysm; however, the thoracic aorta is an uncommon location for a pseudoaneurysm.^5^ Other organisms classically associated with mycotic organisms include *Mycobacterium tuberculosis* and *Treponema pallidum;* however, with routine antibiotic use *Staphylococcus aureus*, *Streptococcus* species, and *Enterococcus* species have become more common.^6^ Fever, anorexia, and chills are common presentations. Left lower quadrant pain is not as common, and a positive result on urine culture prompting a routine callback in a busy ED is most definitely an uncommon presentation for an uncommon disease.

The patient had a very thorough work-up preceding the final diagnosis, including a CT of his chest and the CT of his abdomen and pelvis. However, because the images did not include the area of pseudoaneurysm, his final diagnosis remained unclear until the urine culture provided additional justification to repeat imaging. The time to diagnosis is understandable as this was a rare presentation of a rare disease that was discovered only when *Salmonella* grew where it did not belong.

Difficulty in diagnosing mycotic aneurysms accounts for the high mortality and is due to the vagueness of the initial clinical presentation, as was the case with the patient described. Commonly, patients report days to months of intermittent fevers, fatigue, abdominal pain, nausea, anorexia, or back pain.^5^ Given the rarity of infected aortic aneurysms, case report data describe only standard diagnostic techniques, including CT, echocardiography, and magnetic resonance imaging. Transesophageal echocardiography better delineates thickening of the aortic wall or sequela of dissection.^7^ If targeted antibiotic therapy can occur before aneurysm formation, then mortality may be as low as 25%.^5^ However, if no diagnosis is made until the time of aneurysmal rupture, mortality may exceed 65%.^6^ Historically, mortality rates with medical treatment alone may reach 100%. Therefore, experts agree that treatment should involve surgical replacement of the infected aorta in conjunction with targeted antibiotic therapy.^8^

## ADDENDUM

This case report is offered to the family as an apology for the patient’s delayed diagnosis and a promise to share our knowledge with the medical community to prevent missing this rare diagnosis for future patients. The family was intensely gratified and comforted that our reporting the case might afford a quicker diagnosis for a patient who presents in the same manner.

An apology is a restoration of trust that offers a continued partnership in care. Apologies heal by satisfying one or more of the following values: restoration of self-respect, assurance of shared values, elimination of self-blame, preservation of safety in a relationship, continuing dialogue, and reparation of harm.^9^ Our apology eliminated self-blame for the family, assured shared values and safety in the relationship, and made reparations through commitment between the patient and family and the healthcare providers to broaden dissemination of education with hopes of care for other patients.
